# Spatial scale, time and process in mega-events: The complexity of host community perspectives on neighbourhood change

**DOI:** 10.1016/j.cities.2016.01.012

**Published:** 2016-04

**Authors:** Julie Clark, Ade Kearns, Claire Cleland

**Affiliations:** a Urban Studies, School of Social and Political Sciences, University of Glasgow, United Kingdom; b MRC/CSO Social and Public Health Sciences Unit, University of Glasgow, United Kingdom

**Keywords:** Mega-events, Regeneration, Community, Gentrification, Housing

## Abstract

A focus on the ‘mega’ aspect of hallmark events can divert attention from the micro – those local communities who are most impacted by the event. Similarly, attention to the ‘event’ aspect underplays the long process of bidding and preparation before any putative legacy of urban transformation for local people. This paper uses qualitative data to unpack the complex and multi-layered views of local residents, living in a deprived neighbourhood beside the Glasgow 2014 Commonwealth Games site in Scotland. They reflect on five years of intensive urban regeneration, evaluate the experience of ‘lockdown’ at Games time, and consider their hopes and fears for the future of the community. Interviewing a mixture of lifelong, established, new and returning residents, we found considerable common ground across the different groups in terms of hopes for a new, mixed community in the area. However, findings also highlight concerns around urban governance practices and the limitations of a market-led approach to regeneration.

## Highlights

• Host communities experience a mega-event as a process with strong emotional impacts.
• Perspectives were varied and nuanced, rather than binary ‘for or against’.
• Participant views varied between pre-event, during and post-event time phases.
• New housing was welcomed but there are concerns about future management.
• The analysis highlights concerns about market-based processes of change.

## 1 Introduction

### 1.1 Mega-events and the micro-geography of impacts

Mega-events can be considered an integral component of much 20th century urban development (Muñoz, 2006), with urban transformation and ‘legacy benefits’ used to justify the expenditure (Essex and Chalkley, 1998, Leopkey and Parent, 2011, Pound, 2003, Smith, 2012). In many ways, the essence of a mega-event is scale. Müller (2015) argues that there are four integral dimensions, along which scale should be considered: visitor attractiveness; mediated reach; cost; and transformative impact. Hosting a mega-event has been described as ‘one of the most fundamentally political acts of the modern age’ (Horne and Whannel, 2012: 204) which, necessarily, advantages some and disadvantages others. Indeed, securing and delivering a mega-event speaks to the power of the host city's elite (Liao and Pitts, 2006).

As Müller's framework demonstrates, the criteria that define a mega-event predominantly involve macro-scale interests. Reflecting this, evaluation data and impact assessments generally use aggregate monitoring data at city, regional or national levels eliding uneven development and differential community-level experiences (Kasimati, 2003, Preuss, 2004, Owen, 2005). Furthermore, well-intentioned, elite attitudes towards the preferences of relatively disadvantaged groups may be based on assumptions rather than knowledge (Ahlfeldt et al., 2012).

However, this paper prioritises a neighbourhood ‘host community’ perspective on the impacts of a mega-event, using the Glasgow 2014 Commonwealth Games as a case study. The ‘transformative impact’ for the built environment and population (Müller, 2015) draws attention to specific geographies, with stadium-building, new ‘village’ accommodation and adapted transport systems having differentiated effects across the city. It is in the host neighbourhood of a mega-event that the most dramatic impacts can be seen and where, despite a promised legacy, the interests of those promoting and managing the event do not necessarily align with those of those most affected by it. Although urban regeneration and (positive) legacy have increasingly been used as a rationale for undertaking large sporting or cultural events, the question of ‘who benefits?’ is still germane (Coaffee, 2012, Essex and Chalkley, 1998, Hall, 1992).

This paper argues that neighbourhood-level geography and time are as salient as ‘mega’ scale in understanding the nature and impacts of a mega-event. We begin by examining the grounds on which the validity of event-led regeneration has been challenged from the host community perspective. Following this, the Glasgow 2014 Games (GCWG), the study area and research sample are introduced. An exploration of local residents' perceptions of the nature and drivers of change in their neighbourhood, their attitudes towards GCWG regeneration, and their fears and ambitions for the future is used to illuminate the case. The paper closes with reflections on the limitations of mega-events as a catalyst for regeneration and the scope for sustainable, mixed community development around the regeneration site in the post-event era.

## 2 Host community impacts: the mega-event as process

It is tempting to think of the mega-event as a point in time. However, for the host community, from bid, to event, to aftermath, it is more appropriately considered as a process. Physical space is reshaped in order to accommodate the event and social relations are reconfigured, influencing how local people and place are understood, by themselves and by others (Gaffney 2010). From this perspective, time, as well as geography, is a crucial factor in considering the community impacts of a mega-event. We consider the existing literature around three phases, with the host community seen as disadvantaged in each case.

### 2.1 Pre-event: physical displacement and development

The pre-event phase comprises decision-making about the event and associated regeneration, with displacement and demolitions preceding the construction of new infrastructure. Here, the host community often finds itself in opposition to planned developments, being seen as an entity to be managed or maligned in order to facilitate the delivery process.

Despite the high profile of legacy, the language of policy planning can serve to obscure both agency and who benefits (Marcuse, 2015). Although ‘the city’ is identified as bidding for and hosting a mega-event, in the triumvirate of the state, community and capital, it is the community that has the weakest voice. High economic stakes and fixed deadlines systematically militate against successful democratic participation, and elite actors rather than local communities drive the urban development agenda (Garcia, 2004, Hayes and Horne, 2011, Surborg et al., 2008).

From the organisers' perspective, community involvement constitutes a ‘risk factor’, which can interfere with project delivery, while resistance to demolitions or development is construed as not ‘business-friendly’, and often suppressed or downplayed (Raco 2014: 183; 194; Lenskyj, 2002). Fussey and colleagues note a pattern of public engagements taking place in a limited form and ‘only after key decisions have been taken’ (2011: 238). Community actors hoping to influence events require the capacity to identify and engage assertively with layers of complex and changing bureaucratic structures at multiple levels (Olds, 1998, Armstrong et al., 2011). In rare cases, pre-event host communities have strong legal support (Sadd, 2010). More commonly, they are heterogeneous and relatively disadvantaged – by virtue of economic resources, youth, minority or migrant status – when it comes to forming coalitions and lobbying to defend their position (Hall, 2005, Shin and Li, 2013, Smith and Himmelfarb, 2007). Some marginalised groups have made effective use of media coverage to advocate for their interests, lobbying on compulsory purchase, housing, employment and pay (Fussey et al., 2011, Sant et al., 2013, Vigor et al., 2004). Nevertheless, such resistance faces a massive challenge.

The interests of the urban growth machine are enforced through exceptional planning powers, authorising demolitions and the displacement of people (Andranovich et al., 2001, Boykoff, 2014, Gray and Porter, 2014, Hillar, 2003, Lenskyj, 2000). Property developers and owners, including the relatively affluent middle classes, tend to be prioritised by local politicians and media over those of generally poorer, local communities (Gruneau, 2002). Although protecting vulnerable communities or ultimately offering improved social or affordable housing may be the stated aim, some commentators have analysed exceptional planning powers as a manifestation of neoliberal urbanism, with class- or ethnically-based ‘cleansing’ enforced as a prelude to the marketisation of urban space (Harvey, 1989, Kallin and Slater, 2014, Nam and Seok, 2008, Souliotis et al., 2014, VanWynsberghe et al., 2013). Within this framework, the mega-event is an ‘alibi’ (Boykoff, 2014), facilitating a gentrification process: less desirable urban residents can be moved out; more affluent residents moved in; increases in land and property value are captured (Lauermann, 2015, Watt, 2013). Policy and media narratives can be complicit in this process, systematically stigmatising already vulnerable people and places and framing the regeneration intervention as the solution to a problem which they have defined (Gray and Mooney, 2011, Thompson et al., 2013).

Pre-event activity around venues and the Athletes' Village typically includes displacement of resident populations and housing demolition, to the extent that forced evictions are an expected part of the process (Olds 1998), and have been identified in relation to 32 different mega-events since 1980 (COHRE, 2007). Environmental improvement projects prior to the Beijing 2008 Olympics were described as ‘a euphemism for demolition and displacement’, with an estimated 1.5 million people displaced (Centre On Housing Rights and Evictions (COHRE), 2007, Shin and Li, 2013: 559). In other instances, environmentally protected land has been reclassified, and smaller businesses are particularly vulnerable to clearance (Follman, 2015, Raco and Tunney, 2010). The ‘host community’ at the time of the event may, therefore, be markedly different from the community in existence at the time of the bid.

### 2.2 Event time: experiencing securitisation

At the event time, a growing list of behaviours or processes have been defined as security concerns, requiring surveillance or control, with this ‘securitisation’ meaning that living around a mega-event is increasingly likely to involve constraints (Bennett and Haggerty 2011:4; Fussey et al., 2011, Samatas, 2011). Presenting a carefully sanitised image of the city for television audiences, international visitors and corporate interests is an established phenomenon in mega-events, leading to concerns about infringement of civil rights and the impact of the security agenda upon local communities (Graham, 2012, Hall, 2005, Houlihan and Giulianotti, 2012, Newham Monitoring Project (NMP), 2013). Additional to pre-event evictions, ‘cleaning operations’, harassing or detention of minority groups including the homeless, migrants, street children and gay people, have been recorded in numerous host cities (COHRE, 2007).

A military presence within the host community may also be required (Fussey et al. 2011). Security for the London Olympics was boosted to include 18,200 military personnel (more than were deployed in Afghanistan at the time), additional to police and private security services, with the armed forces comprising up to half of security staff in and around the Games venues (Corera and Heald, 2012, Houlihan and Giulianotti, 2012). The Newham Monitoring Project (NMP), a community based civil rights organisation in one of the London 2012 host boroughs, documented a ‘climate of fear’ associated with the intensive security around the Games, which included missile launchers, fighter jets and helicopters overhead, an 11-mile long electrified fence and additional electronic surveillance (Newham Monitoring Project, NMP, 2013: 2).

### 2.3 After the event: economic displacement

In the post-event period, the host community can find that the promised legacy is targeted towards more affluent incomers and non-residents. The main economic benefits from the event may be felt in other parts of the city. Where there are jobs for local people, these might be temporary or of poor quality, leading to frustration and disappointment within the host community (Boyle et al., 2008, Gray and Mooney, 2011, Newman, 1999). Mega-events are said to function as a means of gentrification that ‘permanently place housing beyond the financial means of a significant segment of society’ (COHRE, 2007: 11). The mega-event village is an embodiment of the ‘new’ urban image, the housing generally being for new, more affluent people (Muñoz, 1997, Sadd, 2010). Furthermore, there can also be a ‘ripple’ effect in housing costs beyond the Village, with rising land and property prices resulting in increased rental costs, pricing out those on low incomes (Sadd, 2010, Pillay and Bass, 2008). Host community members can be further marginalised as familiar neighbours leave, support services close, and older businesses or social hubs are replaced by amenities more appropriate for the new, more affluent community (Marcuse, 1985: 208; Varley, 1992).

‘White elephant’ facilities, appropriate for a major international festival but not sustainable on an ongoing basis, are one of the most notorious risk factors in relation to mega-events (Gold and Gold 2011). However, there is also the possibility that relatively well-used venues may serve only more affluent citizens and not be financially accessible to the host community (Clark and Kearns, 2015). Similarly, transport improvements often favour more affluent members of society, such as drivers or train users.

## 3 The Glasgow 2014 Commonwealth Games

The challenge of evaluating legacy within any given context is exacerbated by the diverse nature of mega-events themselves. This being the case, any consideration of potential legacy, either positive or negative, should avoid simplistic inferences about events which may be qualitatively quite different. Although not one of the largest mega-events, the Glasgow 2014 Commonwealth Games (GCWG) is still of considerable scale and can be considered a critical case through which the potential of a mega-event to benefit the host community might be explored. The host city has well-developed governance and partnership structures with considerable experience of event management and ‘boosterist’ activity (Boyle et al., 2008, Garcia, 2004). From the time of the successful bid in 2007, local and national government worked together with the explicit aim of leveraging the event as a regeneration initiative, in accord with good practice that there be an early focus on legacy, embedded within pre-existing urban strategy (Law, 2002, Matheson, 2010, Vigor et al., 2004); in this case, the event occurred one third of the way into a 25 year regeneration project. In short, should things *not* go well for the host community in this instance, it could be said that there is little hope of positive outcomes in the case of other cities and circumstances.

### 3.1 Dalmarnock: a critical place at a critical time

The host community of Dalmarnock is a historic area of Glasgow 4 km east of the city centre. From the postwar period, Dalmarnock suffered from deindustrialisation, population decline, deteriorating housing stock, and increasing unemployment (Barke and Sim, 1981, Rich, 1981). By 2012, after the demolition of four tower-blocks a decade earlier, the area included around 700 households, mostly social renters, living amidst considerable vacant and derelict land. Relative poverty and poor health have led to stigmatising media coverage of Dalmarnock as a ‘problem place’ inhabited, by extension, by problem people (Mooney, 2009: 442).

**Fig. 1:**
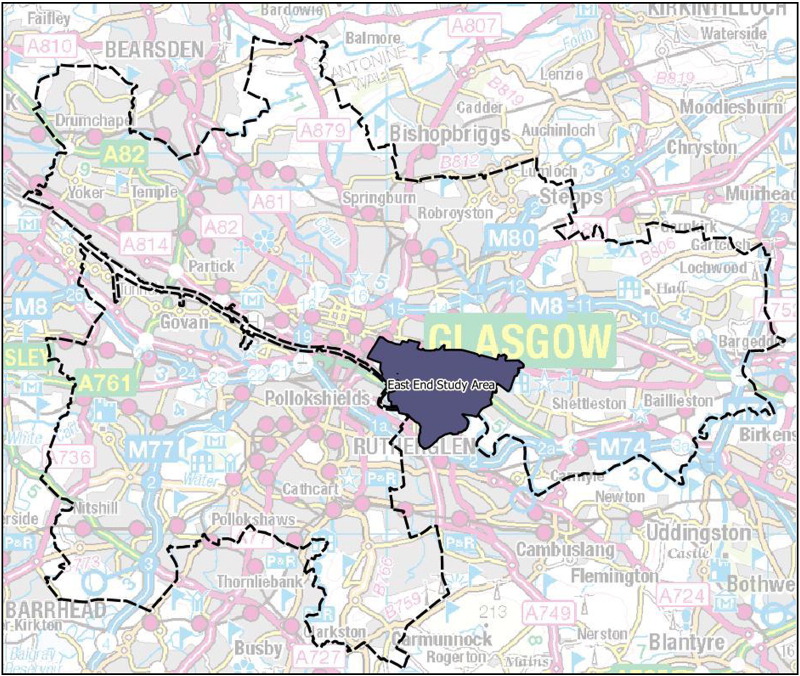
Glasgow City and the wider East End study area.

**Fig. 2:**
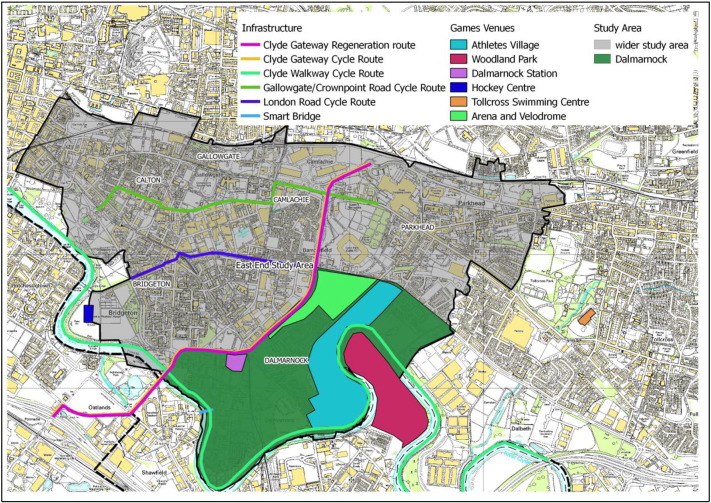
Dalmarnock and Commonwealth Games-related regeneration developments.

Dalmarnock is the site of the £230 m Athlete's Village, retrofitted to provide 400 homes for rent and 300 homes for private sale. Flagship venues for the GCWG, the Emirates Arena and Sir Chris Hoy Velodrome (‘Arena’ and ‘Velodrome’) were built to the north-west of the area (See Fig. 1, Fig. 2). Land clearance practices in the pre-Games period have been criticised, since private land owners were compensated on the basis of potential land value following development while private householders received only current property value (Gray and Porter, 2014). The high-profile eviction of one owner-occupier resident (Mrs Jaconelli) placed much media attention on the area. The family concerned was isolated for five years during a dispute over compensation, and after neighbours in social rented accommodation were moved out of their housing block (Porter et al., 2009). The block previously included a Post Office and the last local shops in the area, which were lost to the community.

## 4 Research aims and methods

Focusing on Dalmarnock, where the greatest level of intervention in relation to the 2014 Commonwealth Games took place, we sought to explore host community experiences and perceptions in relation to:

• Reflections on the past
• Views around the present
• Hopes and fears for the future

### 4.1 Data collection

Interviews with Dalmarnock residents were undertaken in May–July 2014, immediately preceding the GCWG. The timing was planned to capture experiences of living with a mega-event before the event itself coloured perceptions. Given the substantive relevance of gentrification processes, interviews were conducted with people responsible for paying the rent or mortgage, since they would have most insight into locational decisions. Initial contact was made by letter, approaching social housing residents in the area and others who had previously participated in social research. In order to take account of the changing community profile since the beginning of the regeneration, we used a snowball sampling method to engage residents with a variety of knowledge of, and perhaps, commitment to the area (Table 1).

**Table 1:** The research sample.

Category	Description	Pseudonym
Long-term Residents: Resident in the area from before 2007	Life-long: Lived only in Dalmarnock. (n = 2)	Barbara
		Fiona
	Established: Long-term Dalmarnock resident. Also, periods of residence elsewhere. (n = 10)	Angus & Iain (son)
		Donna
		Alan & Maria
		Jon
		Linda
		Eleanor
		Alex
		Deborah
		Jim
		Sandra
Recent Residents: Moved to Dalmarnock from 2007 onwards	Returning: Recent Dalmarnock resident. With some previous residence in Dalmarnock or the east end of Glasgow. (n = 4)	Susan
		Mark
		Anne & Dave
		Rob & Cindy
	New: Recent Dalmarnock resident. No previous Dalmarnock or east end residence. (n = 5)	Lucy
		Marion
		Jeff
		Michael
		Catriona

‘Long-term’ residents had lived in the area for at least the last 7 years, since the advent of Clyde Gateway and the award of the GCWG. This group comprised ‘lifelong’ residents, who had always lived locally and other ‘established’ residents, who lived in other areas before moving to Dalmarnock. ‘Recent’ residents were either ‘new’, with no previous residence in Dalmarnock or east Glasgow, or ‘returning’ interviewees who had moved to Dalmarnock within the past 7 years but had prior knowledge of the area, having lived previously either in Dalmarnock or elsewhere in east end of the city.

Semi-structured interviews were conducted, exploring resident attitudes, perceptions and experiences while retaining a level of comparability between accounts (Arthur and Nazroo, 2003). Twenty-five research participants from twenty one households in the area were asked to reflect on community change in the time that they had lived in the area, on changes associated with regeneration and the GCWG, and their expectations of the future.

## 5 Reflections on the past

Reflections on the nature and causes of neighbourhood change explored residents' perceptions of the role of the GCWG. As illustrated below, interviewee perspectives on change in Dalmarnock were informed by their level of familiarity with the area and by age, with a clear division between the *new* resident group and other participants. Following this, neighbourhood composition, displacement and loss of amenities are explored under the theme of *demolition and its consequences*.

### 5.1 New impressions

The *recent residents* were consistently positive about regeneration and the GCWG, focusing on the new or improved sports facilities in the area and the refurbished train station, which had recently reopened. For this group, regeneration and the GCWG were an attraction to the area. Lucy and her husband bought their home, regarding the area as ‘up and coming’, intending to let the property in the future if they were to move. Jeff and Michael were both social renters, pleased with the quality of their housing association properties as well as the change in the area. Marion owns a home in a mixed-tenure development and though change had not yet gone far enough. She was disappointed by the failure of a proposed hotel and retail development on vacant ground close to the Arena. Although resolute that she lived in a working class area and that the nature of the area was not starting to change (‘Not at all’), Marion would prefer:

…improved transport facilities, doctors surgery and pharmacy, more of a choice of normal shops, getting rid of the pathetic pubs, a hotel or sort of bar that did, was more middle class. (Marion, *new*).

Michael (*new*) agreed that more should happen, with the proviso that local people ‘buying in’ was crucial. Despite the association of gentrification and ‘pacification by cappuccino’ (Zukin, 1995: 28), these sentiments seem quite far removed from revanchist urbanism (Smith, 1996). Nevertheless, Lucy thought about changes in people as well as the environment, saying there were fewer ‘scummy people and neds^1^’ hanging around before saying she felt safer in the area, partly connected to increasing familiarity and making friends in the neighbourhood. Optimism about the neighbourhood's future and a sense that physical changes have made the area look ‘less depressing’ were the dominant emotional impressions given by *recent residents*.

### 5.2 The long view: signifiers and drivers of neighbourhood change

Despite the intensity of recent demolition and construction activity, only three of the *long-term residents* focused on the GCWG as the main driver of neighbourhood change, with the rest having divergent perspectives on how, when and why their neighbourhood started to change.

Most *long-term* interviewees identified physical factors as the primary signifier of change in Dalmarnock, particularly the demolition of social housing some years before the GCWG bid. Eleanor and Alan (both *established*) described cycles of demolition and building, including the refurbishment and subsequent demolition of social rented homes in the early 2000s. Given these experiences, it is easy to see how local residents might be skeptical of potential legacy.

Fiona, (*lifelong*) suspected that earlier property demolition in the area may have been connected to later developments. Added to this was the perception that changes were ‘not for the benefit of the community’ (Jeff, *new*) and that ‘a lot of people are getting their pockets lined’ (Barbara, *lifelong*).

However, de-industrialisation from the 1960s onwards was considered the driving force of neighbourhood change by some older residents: as industry closed down, people followed employment opportunities and, as part of a slum clearance programme, much of the population was moved to peripheral housing estates. For these participants, the GCWG is therefore understood within a historical context of heavy industry and poor housing in an area that was ‘Black, grimy, foggy. Always dark and foggy. A lot of smells…’ (Cindy, *returning*).

### 5.3 Demolition and its consequences

Demolition and its consequences formed the other major theme, as participants reflected on neighbourhood change.

#### 5.3.1 Neighbourhood composition

Many interviewees identified the demolition of high flats in the period before the GCWG bid, as a pivotal time for the area. Such large-scale clearance is generally believed to be damaging to communities (Paris and Blackaby, 1979). However, participants here were supportive about the demolition of the flats and the subsequent relocation of residents, believing it had changed the composition of the neighbourhood for the better. Alex associated the demolitions with what might be considered ‘cleansing’ practices common in the run up to a mega-event (Grix, 2013), moving out ‘hooligans…all the troublemakers’ (Alex, *established*). However, for one couple, this displacement, alongside regeneration activity, attracted them back to neighbourhood after living elsewhere:

That's why we decided to come doon here because we heard they were making it a better area an’ they threw a’ the riff raff oot.

(Dave, *returning*)

The dismissive language above could be considered as ‘distancing’ language, used by the speaker to differentiate themselves from stigmatised populations or places (Wacquant, 2007a). However, other interviewees were more delicate in communicating the same idea while speaking of the neighbourhood being quieter and safer.

It seems, at least from the perspective of those that remain, that disorder rather than poverty or class may have been at issue and there was consensus over the neighbourhood now being a better place to live than 15–20 years ago. Furthermore, Dalmarnock has attracted both the *new* interviewees and *returning* participants who either have knowledge of the area through previously living there, or are returning residents after time elsewhere, seems to reinforce the point.

#### 5.3.2 Displacement

With regard to more recent GCWG-related social change, two interviewees mentioned the Jaconelli eviction. Despite acknowledging that ‘fair enough, it was her family home’, on balance, Michael (*new*) took the view that ‘those flats down there had had their day. It was time to go’. For Jon (*established*), the heavy-handed nature of the eviction, with ‘over a hundred police to get rid of her and her husband from one flat’, provoked a distrust of the authorities involved:

I mean, you know, if they do all that stuff under the glare of publicity, you know? (Jon, *established*)

Jim and Susan both belong to Show People communities,^2^ who have longstanding ties with Dalmarnock. Many were displaced by recent regeneration and now live in other areas, some abandoning their traditional lifestyle. Susan (*returning*) had to relocate but managed to stay within the area. She was unhappy with the reason given for the relocation, that caravan sites would be an ‘eyesore’ for tourists. In the case of Jim's site, pressure to relocate seems to have been successfully resisted:

We, like anybody else, kicked up a lot of stink, and one thing and another, finish up, not one of us has moved.

(Jim, *established*)

While Mrs. Jaconelli's eviction did not feature strongly in resident accounts and ultimately some of the Show People have secured a long-term future in the area, these aspects of displacement reinforce concerns around mega-events and the treatment of vulnerable and minority groups (Bernstock, 2014, Gray and Porter, 2014, Watt, 2013).

#### 5.3.3 Loss of amenities

The closure of small, local shops and a post office, in order to make way for the new Athletes' Village (‘the Village’), was a widespread concern in the interviews. Although there is a very large supermarket to the south-east of the area, this was considered, by some, to be an inaccessible and expensive option. A consequent need for car transport was emphasised and some interviewees described elderly or less physically able neighbours resorting to taxis, a costly option for people on low incomes. Although, intermittently, there was temporary shopping provided initially, by Clyde Gateway and, later, a local entrepreneur, the value of local shopping to the social life of the area, as well as for practical purposes like the collection of pensions or small, daily shopping needs, came across clearly in the interviews. The loss of local shops, children's play areas and the closure of the Dalmarnock community centre also had negative impacts on socialisation, day-to-day exercise, and building social support in the area.^3^

## 6 Views around the present

There is a tension between the long-term process of regeneration and the fixed deadline of a mega-event. To ask about attitudes towards the event and associated developments in the host community is, necessarily, to ask about the whole cluster of experiences around living with that event. Views on the present, therefore, are divided into reflections on event-related regeneration processes, and experiences of or opinions about the new developments at the time just before the event.

### 6.1 Processes of regeneration and event management

#### 6.1.1 Eleven days of sport, five years of dirt and disruption

The physicality of the regeneration experience is a recurrent theme, recounted directly by established community members and second-hand by more recent residents who had been less affected in the preceding years, but sympathised with their neighbours. In some cases, interviewees were relatively tolerant about disruption, mostly because it was considered a necessary evil:

Changes now, I think, are absolutely horrendous but understandable.

(Barbara, *lifelong*)

They have been... well no’ too much disruptive. It's just there's been a lotta inconvenience.

(Dave, *returning*)

However, two of the established interviewees, Donna and Linda, revised their views on the GCWG from initially supportive to hostile, due to disillusion with the closure of shops and the community centre, increasing disruption and a sense of being disregarded by the authorities.

Demolition, digging up the foundations of old buildings, construction of roads, houses and facilities resulted in noise, vibration and continual dust over a protracted period, causing damage to cars and property, requiring repeated cleaning. Construction lorries were another source of noise and a cause of safety concerns. Alan was concerned about health risks because of contamination from old industrial works being disturbed, saying ‘there's an awful lot of people getting cancer an’ that livin’ down here’ (*established*). For both visitors to the neighbourhood and residents, the amount of physical change in the area was described as ‘disorientating’.

#### 6.1.2 Restrictions and rumours

The disorientating effect was amplified in the weeks before the GCWG, with the erection of security fencing and barriers restricting movement along different roads and pathways, which attracted the most widespread and heartfelt criticisms. Beyond the practicalities of negotiating re-routed transport and around blocked streets or pathways, there were issues of status and respect, including: being on ‘lockdown’, a term otherwise applied to prison governance; being unable to park near your own home; knowing that you are not one of the ‘important’ people who have access to dedicated transport lanes; or being monitored by security guards and cameras and feeling imprisoned:

As I say, the best thing they could've done is just put a big cage right roond the whole lot, gave you a horse's bag and told you to go oot there an’ feed yersel’. That's how good it's got.

(Angus, *established*)

Several participants felt that, recognition of the challenges of living with a mega-event was completely absent beyond the community; Deborah exclaimed ‘if they'd even have paid us any kind of lip service, even’ (established). Interviewees who had been told that they might get a one-off cleaning service or car wash token reacted with derision about the usefulness of the offer. The idea that they were living though a short-term inconvenience caused considerable resentment (Alex, established).

Rumours were a recurrent theme in relation to GCWG security restrictions and there was considerable criticism of the level and nature of communication with residents along with a view that no-one was responsible for coordinating between the GCWG and the community:

Nobody fae the Council are coming and telling us exactly… all they'll tell is ‘see when it's aw finished, you'll no’ know the place’ (Laughs).

(Sandra, *established*)

A lack of clarity over exactly what would happen and what the implications of the restrictions would be for local residents led to anxiety and discontent: some people had heard that there would be a curfew in place, that children could not go out in the street after 6 pm or that playing music after 9 pm would be prohibited in case it disturbed the athletes or that the petrol station would not be able to sell petrol while the GCWG were on. Interviewees experienced uncertainty about being able to get to work and access to health services or the shops, either for themselves or on behalf of their neighbours. Most seriously, in an area including many people with health problems, interviewees spoke of security barriers disrupting ambulance and healthcare worker access.

Some residents mentioned or had attended local meetings put on by the authorities, but there was also anger and frustration that the purpose of these was perceived to be delivering decisions that already been made, rather than listening and making decisions based on local community input. People's experience led to scepticism over the place of the community in ‘the pecking order’ (Deborah *established*) and may have contributed to mixed views about the Games and a polarised atmosphere at meetings. Michael (*new*) described being at a community meeting where ‘at least two people have said to my face ‘this is a fantastic thing’ and at the community meeting shouting ‘bloody Commonwealth Games’. At some level the GCWG may have succeeded in building community capacity in ways not quite anticipated; Linda contributed what might be considered the most positive perspective on meetings associated with the GCWG:

I think wi’ all this it's brought people closer the gither, ‘cause everybody's in the same boat… everybody's got the same anger. You know, at what's happening tae them.

(Linda, *established*)

### 6.2 New infrastructure and amenities

Interviewee experiences and attitudes towards the new infrastructure, amenities and housing were predominantly supportive, although participants who were least supportive of the Games offered surprisingly little commentary on regeneration developments, other than the housing, which tended to be positively regarded.

#### 6.2.1 Infrastructure developments

With regard to infrastructure improvements, the recently reopened train station was praised as providing a good service, being well-used and also feeling safer than before. None of the participants mentioned cycling personally, but Fiona (*lifelong*) was pleased that the new cycle-way provided a safe route for her son to cycle to the new Arena. Others commented on watching people cycling in the area, including across the new bridge across the river Clyde connecting Dalmarnock to South Lanarkshire. Several participants mentioned the new roads favourably. For Lucy (*new*), they were one of the attractions in moving to the area. Catriona (*new*) does not drive but reported that visitors and neighbours had found them a great improvement. However, there was widespread dissatisfaction over ‘shocking’ bus services, which are a more important mode in less affluent communities.

#### 6.2.2 Sport and leisure facilities

Some commentators have questioned the value of the new sport and leisure facilities to the local community, particularly, whether a velodrome is a useful amenity (McCartney et al., 2012). Jeff (*new*) shared the view that the facilities are not really for local people, ridiculing the cost of the Arena spa. Alan (*established*) also thought that the gym and velodrome might be quite expensive but said that a lot of local children used them.

However, aside from the spa component, participants were generally pleased with other facilities, which were considered attractive and well built. *New* and *returning* residents were most likely to enthuse about the developments. Of the newer residents, Michael, Rob and Cindy were particularly enthusiastic, with the latter two having visited all of the new amenities close to the area. Michael described the arena and velodrome as ‘fantastic structures’, as well as mentioning that his daughter and her friends liked to go there to watch the boys playing five-a-side football.

Overall, *lifelong* and most of the *established* residents also considered the facilities positively. Fiona (*lifelong*) said her son went to football classes at the Arena. Jon (*established*), although noting that the new facilities were of less use to older residents, planned to cycle at the Velodrome, while Mark and Deborah also knew family and people in the area who had already used the gym and velodrome. Alex and Deborah, who had been especially critical of some aspects of the GCWG and regeneration, described the Arena and Velodrome as ‘amazing’ and ‘a fantastic asset to the area’. Other interviewees spoke about the new woodland park development across the river as a welcome addition for local children. The redevelopment of the previously derelict ‘Olympia’ building in neighbouring Bridgeton also attracted approval, as did the re-opened library and new mediatheque inside.

#### 6.2.3 The Village

Of all the new developments in the area, the Village attracted most enthusiastic discussion. Considering the GCWG overall, Angus and Linda were amongst the most critical interviewees. Angus was against the city hosting the Games from the beginning, believing money should not have been spent on shops and homes rather than new, prestige sports facilities:

I'd say the Commonwealth Games should've been in Edinburgh because Edinburgh's got it a’ whereas we've not.

(Angus, *established*).

However, he was amongst several interviewees who said that they or their relatives would like to live in The Village if a home became available. Similarly, Linda was pleased to get a leaflet inviting her, along with other neighbours, to go to a viewing of the Village properties. As well as being impressed with the quality of the houses, she was pleased about new homes being built in the area. The aesthetic appeal of the houses was praised as ‘lovely’ and ‘amazing’ as well as ‘high-spec’, and the reuse of what was previously wasteland was a cause of satisfaction.

## 7 Hopes and fears for the future

Hopes and fears for the future clustered around two main themes: overcoming stigma and the sustainability of positive changes in the area; and the extent to which the Village and new amenities would benefit the local community.

### 7.1 Stigma and the sustainability of improvements

A sense of the neighbourhood changing for the better was often grounded in reflections of a more challenging past. Older residents, in particular, spoke about historical deprivation and the impacts of stigma. A ‘blemish of place’ (Wacquant, 2007b: 67), based on the area's reputation for violence, gang fighting and muggings, had become associated with the people who lived there, making it difficult for them to find work:

You just said you came fae somewhere else. Rutherglen, Lower Rutherglen. That was a good one you used to tell…Anywhere but Dalmarnock!

(Alan, *established*)

Several residents shared experiences of previous, casual stigmatisation, as passing strangers and even friends made judgements based on the appearance of the neighbourhood; Linda was not alone in finding it hurtful and unjust that a poorly maintained neighbourhood would reflect badly on anyone who lived there:

You know, it's like, ‘Oh, look at the state of they fences, imagine living in that, it's like Chicago’ and all this, you know? And you don't... no, it's no’ like that. It is nae like that, you know?...You hear them on the bus coming on, “I would nae like tae live in there.” And yet the people are absolutely fantastic…You know, and I think people make a place.

(Linda, *established*)

Area stigmatisation was not entirely relegated to the past. Supporting Gray and Mooney's research (2011), participants criticised the media for projecting a misleading image of the area, conflating low income with moral degradation and ignoring successes. Rob spoke about his daughter, who was brought up in Dalmarnock when he lived there years ago:

She's a nurse. But you don't hear the good things; they were wanting certain people to come down to the Olympia to gie something negative about living in Dalmarnock. I said there, ‘My daughter got a degree. Do you want to hear about that?’

(Rob, *returning*)

However, some interviewee accounts included evidence of stigma reversal prior to the GCWG. Michael spoke about being proud to put ‘Dalmarnock’, rather than just ‘Glasgow’ when writing his address. Similarly, Fiona, one of the *lifelong* residents, said that now she tells people she is from Dalmarnock, when previously she would name a neighbouring area because she had felt Dalmarnock was a bad place to come from, with a reputation as a ‘rough’ area. The more enthusiastic advocates of recent change in Dalmarnock made comparisons with the west end of the city and more affluent suburbs, regarding the regeneration as a means of reducing environmental inequalities.

Interviewees valued higher levels of neighbourhood maintenance and a perception that the area's reputation had improved as a result. Some associated these benefits with the security and surveillance aspects of the GCWG, which were not viewed wholly negatively. New security cameras were associated with lower levels of littering and feeling safer, while the attention paid to street cleansing in advance of the GCWG in the neighbourhood was described as ‘fantastic’:

The only way I can describe it before when I first moved here is dirty…I find it very clean now. To be quite honest with you, it's about 17 days to the Games. The council are nothing short of hoovering that street every day.

(Michael, *new*)

However, there were also fears that investment in and the gains to neighbourhood reputation and pride might be lost following the Games; both the place and the people who live there might again be forgotten. In particular, there was also a perception that private interests would continue to be a priority over the needs and wants of local people. Jim observed of the new river walkway:

After the Games, that walkway, if there's a developer comes and he wants so many square feet, and that walkway's in the road, that walkway will disappear.

(Jim, *established*)

Bearing in mind previous cycles of demolition and construction it is unsurprising that, although people welcomed care and attention to their neighbourhood and improvements to the reputation of the area, there was a far more cautious attitude when considering whether positive changes would be sustained over the longer term.

### 7.2 Community or communities?

Looking to the future, new housing and the return of shops to the area were the dominant themes. There was broad agreement that new homes were needed in the area and several participants also saw the Village, transport infrastructure and the new venues as potential mechanisms for revitalising Dalmarnock:

… it looks lovely and, again, it's just waiting (the new Village area) for those to become full of people and hopefully it will sort of turn around, that Dalmarnock will become more populated and hopefully we'll get more shops.

(Barbara, *lifelong*)

There was some agreement that new roads would encourage business and industry to locate in the area. The new venues were also seen as attractions to the neighbourhood, both for community use and in bringing employment opportunities, offering benefits to social and psychological well-being.

The issue of tenure in the Village was important to participants, with opinion favouring social rented or mixed tenure developments. Alan took the view that, without the GCWG, all of the land would have been sold to developers and used for private housing which:

… would nae have done anything for the community. Know what I mean? A lot of the houses that are bought, people in them go to their work, come hame and that's in.

(Alan, *established*)

However, Jon (*established*) felt that having a private component in the housing mix enabled local people who want to buy a house to stay in the area. Like some of the older participants, he reflected on what the loss of more affluent community members meant for sustaining services in the area when tenure is ‘not representative’:

It affected me, you know, people on a low income that weren't, you know, didn't have loads of money to spend in the area and so... So, certainly, you know, I was strongly in favour of, you know, new build housing coming in that would have enabled... people to stay. People that, you know, had money to spend in shops in the area, to stay and live and, you know, be with their families and all the rest and know in the neighbourhood…

(Jon, *established*)

Rather than understanding the planned mixed community in terms of gentrification (Gray and Mooney, 2011), interviewees with a longer perspective considered a mixed community in Dalmarnock, including high quality housing, as a return to the past:

…this is going to be a better class of people, the way it was then. Because in the red buildings that was starter people, you know? They were training to be teachers, owner-occupiers, so that was like their starter houses, so whether they were training to be engineer or whatever. Once they were finished their apprenticeship they would move out the area but that was their start…

(Cindy, *returning*)

However, there were two qualifying concerns to general support the mixed tenure Village, relating to affordability and the implications of the Village for community cohesion in wider Dalmarnock.

Affordability was raised as an issue in relation to both renting and buying locally. Rob and Cindy (*returning*) were originally on the list to move to rented accommodation in the Village but, although still enthusiastic about what the Village might do for Dalmarnock, they accepted a property elsewhere in Dalmarnock, considering it still high quality but less expensive to rent. Mark (*established*) argued that new properties should *only* be available for social rent, on the grounds that ‘if you can afford tae buy a house, you can afford tae buy it anywhere’, and although, the houses for sale might represent very good value, for families on relatively low incomes they are still unattainable. New residents, Lucy and Catriona both regarded their homes as investments, hoping that their value will ‘improve’. Although Lucy mentioned possibly renting their property out in the future, both she and Catriona also seemed connected to Dalmarnock at a deeper level, engaging with neighbours and spending time in the area.

With respect to future community cohesion, there were concerns about who future tenants might be, in both the private and social rented sectors. Cindy described one housing association as:

… only letting to working couples…or (people with a pension). Well, people that are paying their way, you know? Yeah. Some of them are saying that's discrimination for those people on housing benefit...

(Cindy, *returning*)

However, a lack of any vetting procedures was also a concern, with regard to private tenants in buy-to-let investment purchases and social renters. There were fears that without any controls, residents in the current community could end up living next to ‘a thug’ (Alan, *established*).

The wide variation in housing quality within a small area was seen as a potential strain to relations between people in the Village and those in wider Dalmarnock. Some of the housing stock in the area is still very poor, including properties adjacent to the new Village. Angus talked about cold and dampness, saying ‘the mortuary's far warmer than this house’, and described years of waiting for a new home. There was a sense of inequity that long-term Dalmarnock residents got ‘the very cheapest stuff that they could get’ in housing refurbishments while:

…I′ve had a wee look in the Athletes' Village, and they're fabulous… they're lovely, high spec, you know, really, really, quality stuff and… you know, so that's a wee bit frustrating.

(Deborah, *established*)

Nevertheless, for many interviewees, the defining measure of success with the regeneration will not be the Village, the venues, new employment or the Games themselves, but the return of facilities essential to social interaction in the community, particularly small, local shops.

…a whole new community in and nae facilities.

(Linda, *established*)

## 8 Conclusion

Latouche (2007) has described the phenomenon of the ‘imaginary’ legacy: mythologies of good and bad Games, created by sectional interests advocating for their own version of the ‘real’ legacy. Our investigation of the impacts and legacy of the Commonwealth Games 2014 for Dalmarnock, Glasgow shows that perspectives on legacy for the host community are a matter of scale, time and process.

With regard to scale, the summer Olympic Games can be considered the ultimate mega-event. However, smaller-scale ‘*not-so-mega-events*’ are more commonplace and offer a more feasible opportunity for event-led regeneration for many cities; however, relatively little is known about their impacts (Coates, 2012). A focus on ‘mega’ makes it easy to overlook the ‘micro’ – those local communities who are most impacted by the event. In the case of the Glasgow 2014 Games, local legacy related to the mega-event was defined in terms of improving the physical and social environment of the East End of Glasgow (Glasgow City Council (GCC), 2007, Scottish Government, 2014). Our in-depth research was conducted in what might be considered the ‘core’ host community, Dalmarnock, which was most affected by the Games and associated developments. Even though we have focused on one of the smallest communities in the East End of Glasgow, nonetheless, we find a mixture of residents and a variety of opinions. The complexity and heterogeneity of community views in respect of the mega-event is striking. Both between and within individuals, we did not find a singular outlook on the CWG and regeneration; participant perspectives on the Games and regeneration were nuanced, rather than falling into a binary ‘for or against’ pattern. The process of change attracted frustration and, in some cases, vehement negative commentary, particularly relating to shorter-term constraints at the time of the Games. Simultaneously, new infrastructure, amenities and especially housing were broadly welcomed, including by some participants who were very distressed by the regeneration process.

Time is relevant to studying legacy in a number of respects. A mega-event can be analysed in three distinct phases: the pre-event period when the bid and major preparations take place, such as the construction of new venues, and when, for more recent events, associated legacy programmes are put in place; the intensive event period, in weeks before, during and after the event, when arrangements for security, catering, accommodation and travel are put in place; and the post-event period when new facilities are converted for everyday use, with the continuation of some legacy programmes and monitoring. We encouraged local residents to reflect and found that individuals' views on the Games varied between each of these time phases. Nostalgia and disdain for the past could co-exist in complex patterns with discontent or mixed feelings about the pre-event phase, and, again, with both doubt and optimism for the future. People's horizons of memory coloured their boundaries of expectation: how people understood recent social change, and what they felt about the future, often related to the extent of their own history in the area. While some people attributed social changes to the advent of the Games and its associated developments, those with a longer cognitive time horizon viewed recent events as part of a longer process of social change. Rather than a gentrifying process, where lower income groups are displaced by the more affluent (Smith, 2012, Thornley, 2012), these participants see the future in terms of a return to more mixed community, following a period where disruptive tenants were spatially concentrated through housing mechanisms.

As well as being an event realised in the moment, for the local host community, a mega-event is very much experienced as a process in several regards. Many of the interviews demonstrate emotional as well as physical impacts from living in the highly securitised environment just before the event and that these processes of securitisation are found alienating and disrespectful. While current mega-event literature tends to displacement and social change (Centre On Housing Rights and Evictions (COHRE), 2007, Smith and Derkson, 2002), our interviews suggest that a more pressing issue for residents was inadequate communication, with authorities perceived to dictate, rather than consult on their practices. Another is insufficient recognition or acknowledgement of how onerous long-term co-existence with mega-event regeneration can be, given its intensity and scale. Furthermore, that, for those living with a mega-event, it is not adequate to answer these difficulties by directing residents to look to the future.

This analysis also highlights concerns about market-based processes of change, notwithstanding that the overall programme was initiated by public policy. Although we have found elsewhere that residents in the East End of Glasgow exhibit relatively high levels of perceived community empowerment (Clark and Kearns, 2013), in-depth analysis shows that residents in Dalmarnock feel powerless and concerned about two particular aspects of local change. Firstly, they were unable to prevent the loss of their local shops or secure their speedy return; the latter is dependent upon regeneration providing a suitable market opportunity for a general retailer. Secondly, people are well aware of the rapid growth of the private rented sector in the city (Scanlon et al., 2013), which they see as inadequately regulated, and were concerned to avoid concentrations of disruptive tenants, either in new social housing in the area, or in the private sector, when new owner-occupied housing might transfer to the rented sector.

Earlier mega-event research has provided an important critique of the dark side of legacy, focusing in particular on disadvantage to the already disadvantaged (Porter et al., 2009). However, in common with numerous changing post-industrial areas, Dalmarnock includes long-term residents, living alongside newer, incoming residents, and returning residents. In seeking to avoid assumptions about the different residents who have a stake in the area (Ahlfeldt et al., 2012, Latouche, 2011) we adopted an inclusive approach to sampling. Although the use of mega-events for regeneration is likely to remain contentious, from the broad host community perspective, our findings offer some cause for optimism. As Somerville (2011: 16) observes, ‘strong attachment to place does not have to be expressed through claims of exclusive ownership’, and in fact we found considerable common ground in residents' hopes for the future in terms of a new, mixed community in this case. Nevertheless, interviewees' optimism was qualified by concerns about whether future benefits would be sustained and accessible to all; these were matters on which residents wanted a commitment from those responsible for change.

## Footnotes

1 A pejorative term for young, working class men, stereotypically, wearing sports clothes.

2 Long-established communities of travelling fairground workers.

3 Following the Games, a new community centre was opened, including a pharmacy, dentist and medical practice but there was no replacement local, general store at the time of writing.
